# A Forest Tent Caterpillar Outbreak Increased Resource Levels and Seedling Growth in a Northern Hardwood Forest

**DOI:** 10.1371/journal.pone.0167139

**Published:** 2016-11-21

**Authors:** Danaë M. A. Rozendaal, Richard K. Kobe

**Affiliations:** Michigan State University, Department of Forestry, Natural Resources Building, 480 Wilson Road, Room 126, East Lansing, MI, 48824–1222, United States of America; Charles University, CZECH REPUBLIC

## Abstract

In closed-canopy forests, gap formation and closure are thought to be major drivers of forest dynamics. Crown defoliation by insects, however, may also influence understory resource levels and thus forest dynamics. We evaluate the effect of a forest tent caterpillar outbreak on understory light availability, soil nutrient levels and tree seedling height growth in six sites with contrasting levels of canopy defoliation in a hardwood forest in northern lower Michigan. We compared resource levels and seedling growth of six hardwood species before, during and in the three years after the outbreak (2008–2012). Canopy openness increased strongly during the forest tent caterpillar outbreak in the four moderately and severely defoliated sites, but not in lightly defoliated sites. Total inorganic soil nitrogen concentrations increased in response to the outbreak in moderately and severely defoliated sites. The increase in total inorganic soil nitrogen was driven by a strong increase in soil nitrate, and tended to become stronger with increasing site defoliation. Seedling height growth increased for all species in the moderately and severely defoliated sites, but not in lightly defoliated sites, either during the outbreak year or in the year after the outbreak. Growth increases did not become stronger with increasing site defoliation, but were strongest in a moderately defoliated site with high soil nutrient levels. Growth increases tended to be strongest for the shade intolerant species *Fraxinus americana* and *Prunus serotina*, and the shade tolerant species *Ostrya virginiana*. The strong growth response of *F*. *americana* and *P*. *serotina* suggests that recurring forest tent caterpillar outbreaks may facilitate the persistence of shade intolerant species in the understory in the absence of canopy gaps. Overall, our results suggest that recurrent canopy defoliation resulting from cyclical forest insect outbreaks may be an additional driver of dynamics in temperate closed-canopy forests.

## Introduction

In temperate closed-canopy forests, gap formation and closure are thought to be major drivers of forest dynamics. When a large tree dies and a canopy gap forms, light and soil nutrient levels in the understory increase [1] and release growth of tree seedlings and saplings. After 9–40 years [2,3], canopy gaps close through height growth of saplings or increased branch growth of adjacent canopy trees [4]. Multiple gap events are needed for successful canopy regeneration for most tree species (e.g., [5,6]), but the chance of gap formation in closed-canopy forests is small due to low mature tree mortality rates, with an average gap formation return interval of ~50–200 years [7]. Shade intolerant species benefit most from large canopy gap formation, as these species grow fast in high-light conditions at the expense of low survival in shade, while shade-tolerant species have high survival in shade, but do not grow fast in high light ([8], but see [9]). These differential responses to resource levels across tree species can play an important role in driving long-term patterns in forest dynamics [10,11].

Crown defoliation by insects may also influence understory resource levels and thus forest dynamics in closed-canopy forests, especially if insect outbreaks occur frequently and at a large spatial scale. Forest tent caterpillar (*Malacosoma disstria*), for example, has an outbreak periodicity of approximately ten years [12] in northern hardwood forests in the USA. During the typically 2 to 5-year outbreaks, forest tent caterpillar defoliates most northern hardwood species, except for *Acer rubrum*, early in the growing season [12]. Trees refoliate within a few weeks after defoliation during an insect outbreak, although leaves remain smaller than in non-outbreak years [13]. Canopy defoliation increases understory light availability and sometimes soil water [14] and nutrient levels [15], as a result of reduced water uptake and input of nutrients from insect frass and green leaf fragments to the litter layer [16].

Increased resource levels resulting from canopy defoliation could increase seedling growth during the outbreak or in the subsequent year (e.g., [17,18–21]). A lagged growth response could arise from determinate growth [22,23], i.e., growth in the subsequent year depends on environmental conditions and resource levels in the previous year [24], a pattern exhibited by many northern hardwood tree species. We expect that the strength of seedling growth responses to increased resource levels will differ across tree species, with the strongest responses for shade intolerant early-successional species [25,26].

In this study, we evaluate the effect of a forest tent caterpillar outbreak in 2009 on understory light availability, soil nutrient levels and seedling height growth for six tree species in a hardwood forest in northern lower Michigan, USA. Specifically, we test the following hypotheses: 1) light availability and soil nutrient levels increase during the outbreak; 2) seedling growth increases during the outbreak and subsequent years in response to enhanced resource levels; and 3) growth responses to the forest tent caterpillar outbreak will be stronger for shade intolerant than shade tolerant species.

## Materials and Methods

### Ethics Statement

Permission for field work was given by the U.S. Forest Service through a Special Use Permit, issued by the Cadillac/Manistee Ranger District of the Manistee National Forest.

### Study sites and species

Field work was conducted in the Manistee National Forest (44°12’ N, 85°45’ W), in northern lower Michigan in 12 mixed hardwood stands across a soil-fertility gradient in a glacial landscape [27]. Soil nutrient availability increased from glacial outwash sites, to ice-contact sites, to moraine deposits [27,28], and tree species composition changed across these glacial land forms ([27,29], Table 1). The forest stands were all between 80 and 100 years old and the minimum and maximum distance between two sites was 1.6 and 40 km, respectively. Six sites were affected by a forest tent caterpillar (*Malacosoma disstria*) outbreak from 2008 to 2010 [30]. The minimum and maximum distance between two defoliated sites was 3 and 30 km, respectively. The six defoliated stands were located on nutrient-rich moraine deposits, but differed slightly in soil nutrient content ([27], Table 1). Peak defoliation took place in 2009. Two of the included sites were severely defoliated (complete defoliation of nearly all canopy trees), two were moderately defoliated (about half of the trees/canopy defoliated), and two sites were lightly defoliated by forest tent caterpillar (some leaf area removal, but trees were not completely defoliated; Table 1). In 2008, sites were lightly defoliated by forest tent caterpillar. In 2010, site 8 and 9 were lightly defoliated by forest tent caterpillar and in site 7 *Quercus rubra* was lightly defoliated by gypsy moth (D.M.A. Rozendaal, personal observation). Seedlings of six tree species with varying shade tolerance were included in this study: the very shade tolerant species *Acer saccharum* and *Fagus grandifolia*, the shade tolerant species *A*. *rubrum* and *Ostrya virginiana*, and the shade intolerant species *Fraxinus americana* and *Prunus serotina* [31].

**Table 1:** Description of the six defoliated study sites and species for which seedlings were included. Land form, location, estimated defoliation level (in 2009), dominant species in the overstory (the three most abundant species based on stems ≥ 10 cm diameter at breast height; Baribault et al. [27]), and species for which seedlings were included are indicated. Site numbers generally correspond to the numbering in Baribault et al. [27], except for sites 12 and 13, which were merged into one site (site 12). acru = *Acer rubrum*; acsa = *Acer saccharum*; fagr = *Fagus grandifolia*; fram = *Fraxinus americana*; osvi = *Ostrya virginiana*; piba = *Pinus banksiana*; pogr = *Populus grandidentata*; prse = *Prunus serotina*; qual = *Quercus alba*; quru = *Quercus rubra*; quve = *Quercus velutina*; tiam = *Tilia americana*.

Site	Land form / soil type	Location	Defoliation (2009)	Dominant species (overstory)	Included species (seedlings)
1	outwash	44°14'N, 85°56'W	none	qual, quve, piba	-
2	outwash	44°14'N, 85°57'W	none	qual, quve, quru	-
3	ice contact	44°16'N, 85°53'W	none	quve, quru, acru	-
4	ice contact	44°17'N, 85°53'W	none	quru, acru, pogr	-
5	mesic ice contact	44°19'N, 85°52'W	none	quru, quve, acru	-
6	mesic ice contact	44°12'N, 85°48'W	none	quru, pogr, acru	-
7	poor moraine	44°20'N, 85°29'W	severe	acsa, quru, acru	acsa
8	poor moraine	44°22'N, 85°49'W	light	acsa, fagr, acru	acsa, fram, osvi
9	intermediate moraine	44°15'N, 85°45'W	light	acsa, fagr, quru	acru, acsa, fagr
10	moraine	44°13'N, 85°40'W	moderate	acsa, fagr, tiam	acsa, fagr, osvi, prse
11	moraine	44°13'N, 85°45'W	severe	acsa, fagr, tiam	acsa, fram
12	moraine	44°13'N, 85°45'W	moderate	acsa, tiam, quru	acru, acsa, fram, osvi, prse

### Canopy openness

To assess how light availability in the understory responded to the forest tent caterpillar outbreak, and to quantify defoliation levels in each of the six defoliated sites, we measured canopy openness using hemispherical canopy photos. Photos were taken in June 2009, during peak outbreak conditions, and subsequently in June 2010 and June 2011 in overcast conditions or during twilight every 10 m along a 200 m × 1 m transect, consisting of 200 1-m^2^ quadrats. Photos were taken with a digital camera (EOS 5D Mark II, Canon, USA) and a fish-eye lens (15 mm F2.8 EX DG, Sigma, Ronkonkoma, NY, USA), at 50 cm height in the centre of a 1-m^2^ quadrat. The photos taken in June 2009 were unintentionally overexposed, especially in the sites with light canopy defoliation. Thus, for the two lightly defoliated sites, site 8 and site 9, we used canopy photos taken in September 2009, assuming that no refoliation took place in these sites, which is consistent with our field observations. For the two moderately defoliated sites, site 10 and site 12, canopy openness in June 2009 may be slightly overestimated. Canopy openness during peak outbreak may be underestimated in site 11, as *Tilia americana* trees had already refoliated when photos were taken [32]. Canopy openness was calculated using Gap Light Analyzer software [33]. Percent canopy openness is a good predictor of seedling/sapling growth [34].

### Soil nutrients

We measured concentrations of soil nutrients in all twelve sites during the outbreak in 2009 and in the two subsequent years. Soil cores were collected in July 2009, June/July 2010 and in September 2011. Per site, soil samples were taken every 10 m along a 200 m × 1 m transect (200 1-m^2^ quadrats). Three cores of 15 cm length, excluding the thin leaf litter layer (~2–3 cm in summer), were collected, composited and air-dried per sampled 1-m^2^ quadrat. Total inorganic nitrogen was extracted in 2 M KCl solution. Concentrations of nitrate and ammonium were determined with a colorimetric assay using an absorbance microplate reader (ELx808 Absorbance Microplate Reader, BioTek Instruments Inc., Winooski, VT, USA). An ammonium salicylate and ammonium cyanurate colorimetric method was used for ammonium [35] and vanadium (III), sulfanilamide and N-(1-naphthyl)-ethylenediamine dihydrochloride for nitrate [36]. Calcium, potassium and magnesium were extracted in Mehlich III solution [37] and concentrations of total extractable calcium, potassium and magnesium were determined with ICP spectrometry (Optima 2100DV ICP Optical Emission Spectrometer, Perkin-Elmer, Shelton, CT, USA). Instrument detection limits were wavelengths of 393.366 nm for calcium, 766.490 nm for potassium and 285.213 nm for magnesium. We included both soil nitrate and ammonium, as well as their sum (total inorganic N) in the analyses.

We regarded soil nutrient levels in 2011, two years after the outbreak, as representative of non-outbreak values. To evaluate the effect of the forest tent caterpillar outbreak on soil nutrient levels, we compared non-outbreak levels with levels in 2009 and 2010 in the six defoliated sites. Per sampling point, the change across years in 2009 and 2010 was expressed relative to the average change in the six non-defoliated sites over the same years (∆*DEF*_*t*_):

$$\Delta DEF_{t}=\frac{DEF_{t}/DEF_{2011}}{(\sum \left(NDEF_{t}/NDEF_{2011}\right))/n}$$ (1)

where *DEF*_*t*_ indicates a soil nutrient level in a defoliated site in year *t*, while *NDEF*_*t*_ indicates a soil nutrient level in a non-defoliated site in year *t*. Thus, a relative change > 1 indicates an increase in a soil nutrient compared to non-outbreak conditions. As such, we accounted for possible differences in soil nutrients as a consequence of the variation in timing of soil sampling and climatic fluctuations across years.

### Seedling growth measurements

In late August-October 2009, after terminal buds had been set, we selected seedlings for each of the six species, distributed across the sites where that species occurred. In each site, seedling establishment and mortality have been monitored in a 200 m × 1 m transect, thus in 200 1-m^2^ quadrats, since 1998. To evaluate pre-outbreak growth, we included only seedlings that were at least 1 year old (ranging from 1–6 years) in 2008, based on previous seedling censuses. We minimized effects of seed reserves on growth by excluding the first year of height growth. In total, we randomly selected a maximum of 450 seedlings per species, but for species with low abundance all seedlings were selected. We measured height growth (leader extension growth) and total height (length of the leader) to the nearest mm, and estimated and recorded the level of defoliation and dieback for each seedling. For 2010–2012, we repeated the same measurements, after terminal buds had been set. For 2008, we reconstructed annual height growth using terminal bud scars on the leader. Only seedlings that were still alive in 2011 were included in the analyses, leading to a sample size of 32–212 seedlings for the six species. Any height growth interval in which dieback of the leader occurred was excluded from analysis.

### Statistical analysis

We compared canopy openness between 2009, 2010 and 2011, using a linear mixed-effects model. Site and year were included as fixed effects, and we included a random intercept for each 1-m^2^ quadrat in which canopy photos were taken. We compared five models that differed in their fixed effects, using maximum likelihood estimation: (1) site, year and their interaction as fixed effects; (2) site and year; (3) year; (4) site; and (5) just the intercept. Models were compared based on Akaike’s Information Criterion (AIC_c_), adjusted for small sample size. We considered models that were within less than two AIC_c_ units from the minimum AIC_c_ as equally supported [38]. Canopy openness was log_10_-transformed prior to analysis.

To assess whether soil nutrient levels increased in response to the forest tent caterpillar outbreak, we compared the mean relative change in a soil nutrient from non-outbreak conditions (2011) to peak outbreak (2009) and to one year post-outbreak (2010) across the six defoliated sites (∆*DEF*_*t*_) using maximum likelihood estimation. Site-level means and their two-unit support intervals were estimated for all models using a global optimization approach through simulated annealing [39]. Two-unit support intervals are roughly equivalent to 95%-confidence intervals [40]. Relative changes in soil nutrients were log_10_-transformed prior to analysis to enhance normality and homoscedasticity. Thus, if two-unit support intervals included zero, the relative change in soil nutrient levels resulting from the forest tent caterpillar outbreak was not significant.

To assess whether seedling height growth (in mm yr^-1^) changed in response to the forest tent caterpillar outbreak, we compared height growth across years and sites for each species, using linear mixed-effects models. We included site and year as fixed effects, as well as seedling height at the start of the growing season to account for increasing seedling growth with seedling height [41,42], and we included a random intercept for each seedling. Per species, we compared 17 models varying in fixed-effect structures, ranging from a full model with site, year, initial seedling height and all two-way interactions, to an intercept-only model. We calculated the marginal *R*^2^, the variance explained by just the fixed effects, and the conditional *R*^2^, which indicates the variance explained by both fixed and random effects [43]. By fitting species-specific models we did not statistically test for differences in growth responses across species or functional groups (shade tolerance).

We were unable to assess which resource(s) drove growth responses to the forest tent caterpillar outbreak, as canopy openness during the outbreak was strongly correlated with pre-outbreak (baseline) levels of calcium and total organic nitrogen, across all seedlings of five of the six species (Pearson’s *r* = 0.55–0.83). Thus, it was not possible to model height growth in 2009, and 2010, as a function of a combination of non-outbreak resource levels, and/or changes in resource levels. All analyses were performed in R version 3.0.3 [44]. Linear mixed-effects models were performed in the ‘lme4’ package [45]. We used the simulated annealing algorithm implemented in the ‘likelihood’ R package for estimating parameters and two-unit support intervals [46].

## Results

### Changes in canopy openness and soil resources

**Fig 1:**
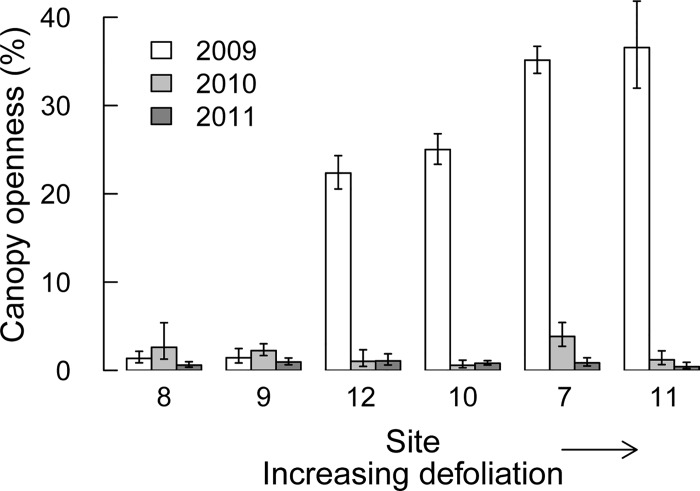
Back-transformed mean canopy openness (± SD) from 2009 to 2011 for six sites that were defoliated by forest tent caterpillar in order of increasing defoliation (*n* = 20 per site, per year). Canopy openness in 2011 was regarded as representative of non-outbreak conditions.

Canopy openness changed across years, as a model with both site and year, and their interaction, performed best. Canopy openness increased strongly during the forest tent caterpillar outbreak in 2009, from 1–2% in a non-outbreak year to over 20% in the moderately defoliated sites 10 and 12, and to over 30% in the severely defoliated sites 7 and 11 (Fig 1). In site 11, canopy openness during peak outbreak in 2009 was likely underestimated, as most *Tilia americana* trees had (partly) refoliated when canopy photos were taken. During the minimal outbreak in 2010, canopy openness was also slightly higher than during non-outbreak conditions (2011) in four of the six sites (Fig 1).

**Fig 2:**
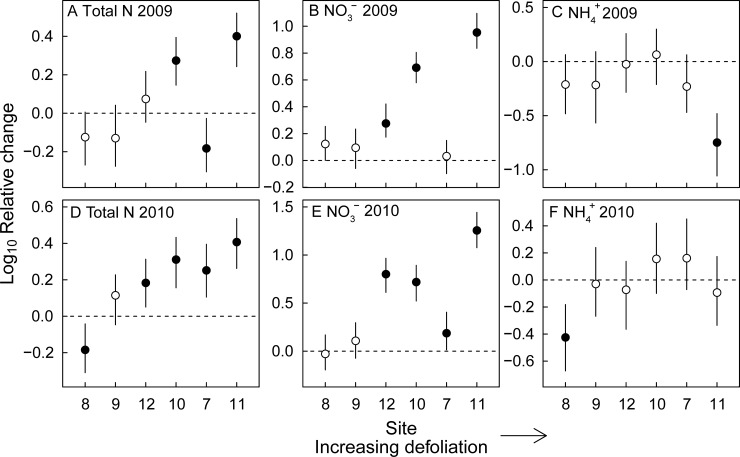
Predicted log_10_ relative change in total inorganic soil N, soil nitrate (NO_3_^-^) and soil ammonium (NH_4_^+^) levels with two-unit support intervals in the peak outbreak year (2009) and the year after the outbreak (2010) relative to the non-outbreak year (2011). Sites are ordered by increasing level of defoliation. Changes were standardized by the average change observed in the six non-defoliated sites to account for annual climatic and other differences. Significant changes across years are indicated by deviations from zero: filled symbols indicate significant changes, open symbols indicate non-significant changes. A-C) Log_10_ relative change from non-outbreak conditions to 2009 (*n* = 11–19, per site); D-F) Log_10_ relative change from non-outbreak conditions to 2010 (*n* = 17–21, per site).

Total inorganic soil nitrogen significantly increased in 2009, during the forest tent caterpillar outbreak, in the moderately defoliated site 10 and the severely defoliated site 11, but decreased in the severely defoliated site 7 (Fig 2A). The increase in total inorganic nitrogen was largely driven by a strong increase in soil nitrate, which significantly increased in the moderately defoliated sites 10 and 11, and in the severely defoliated site 12 (Fig 2B). Soil ammonium did not change, or decreased in 2009 compared to non-outbreak levels (Fig 2C). In 2010, one year after peak defoliation, total inorganic soil nitrogen significantly increased in the moderately defoliated sites 10 and 12, as well as in the severely defoliated sites 7 and 11 (Fig 2D). Similarly, the increase in total inorganic nitrogen was largely accounted for by a strong increase in soil nitrate (Fig 2E), while ammonium levels did not increase (Fig 2F). In both 2009 and 2010, the increase in total inorganic nitrogen tended to become stronger with increasing defoliation of the sites (Fig 2A and 2D). Soil calcium, potassium, magnesium levels did not increase in response to the forest tent caterpillar outbreak (results not shown).

### Seedling height growth responses to the forest tent caterpillar outbreak

**Fig 3:**
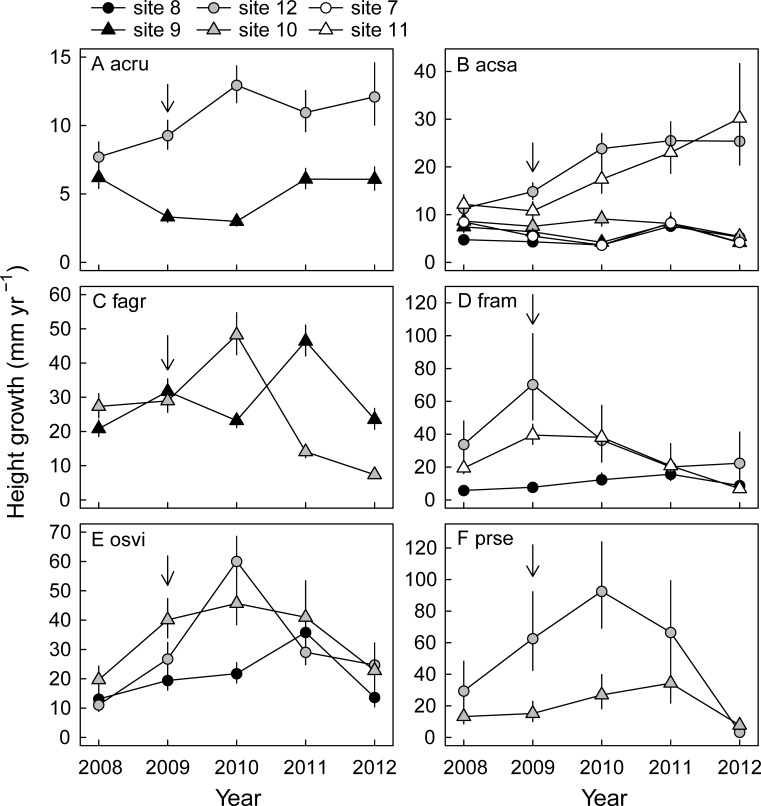
Predicted seedling height growth for seedlings of six northern hardwood species in response to a forest tent caterpillar outbreak. Back-transformed mean (± SE) predicted height growth per site, per year, while holding seedling height constant at a species-specific average across all sites and years. The outbreak occurred in 2009. Note the different scales on the y-axis. A) acru = *Acer rubrum* (*n* = 22–32, per site); B) acsa = *Acer saccharum* (*n* = 16–59, per site); C) fagr = *Fagus grandifolia* (*n* = 34–53, per site); D) fram = *Fraxinus americana* (*n* = 9–62, per site); E) osvi = *Ostrya virginiana* (*n* = 9–13, per site); F) prse = *Prunus serotina* (*n* = 13–21, per site).

**Fig 4:**
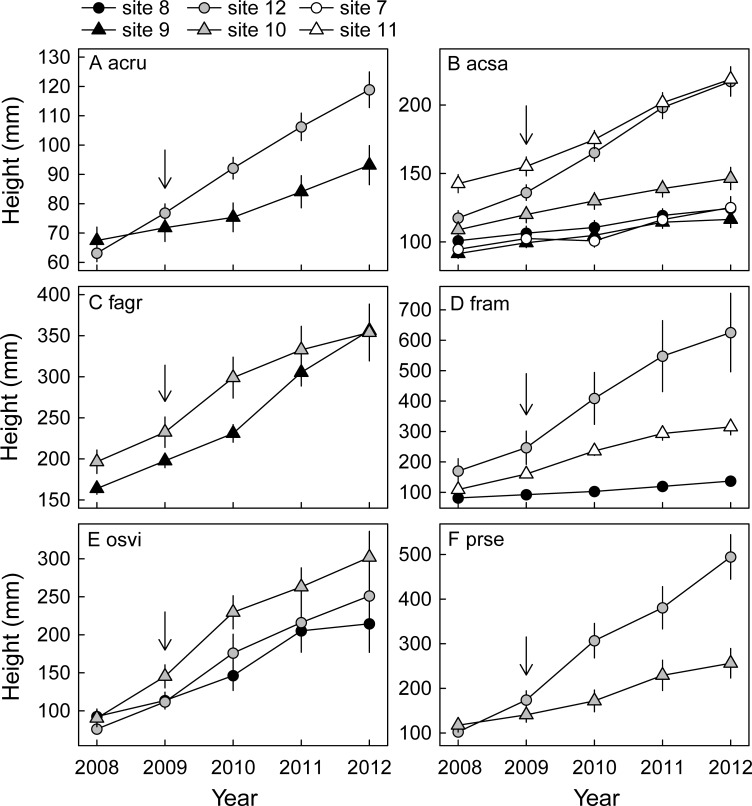
Average seedling height (± SE) per site, at the start of each year, for seedlings of six northern hardwood species. The outbreak occurred in 2009. Note the different scales on the y-axis. A) acru = *Acer rubrum* (*n* = 22–32, per site); B) acsa = *Acer saccharum* (*n* = 16–59, per site); C) fagr = *Fagus grandifolia* (*n* = 34–53, per site); D) fram = *Fraxinus americana* (*n* = 9–62, per site); E) osvi = *Ostrya virginiana* (*n* = 9–13, per site); F) prse = *Prunus serotina* (*n* = 13–21, per site).

For all six species, height growth increased in response to the outbreak in the peak outbreak year, 2009, and/or in the following year, 2010, in some of the sites (Fig 3, Table 2). *Acer rubrum* increased growth in the moderately defoliated site 12 in 2010 only, but did not change growth in the lightly defoliated site 9 (Fig 3A). *Acer saccharum* increased growth in site 12 and in the severely defoliated site 11 in 2010, but did not respond in any of the other sites (Fig 3B). *Fagus grandifolia* increased growth in the moderately defoliated site 10 in 2010, but not in the lightly defoliated site 9 (Fig 3C); growth in 2011 did increase in the lightly defoliated site 9. *Fraxinus americana* strongly increased growth in 2009 in both site 12 and in site 11, and growth remained high in 2010 in site 11, but growth did not change in the lightly defoliated site 8 (Fig 3D). *Ostrya virginiana* increased growth in the moderately defoliated sites 10 and 12 in 2009 and 2010, but not so in the lightly defoliated site 8 (Fig 3E). *Prunus serotina* increased growth in both 2009 and 2010 in site 12, but did not respond in the moderately defoliated site 10 (Fig 3F). Thus, growth responses varied across species. Results of species-specific models indicated that (absolute) growth increases tended to be strongest for the shade intolerant species *F*. *americana*, *P*. *serotina* and the shade tolerant species *O*. *virginiana* (Fig 3). Growth only increased in moderately and severely defoliated sites, but growth increases did not become stronger with increasing site defoliation. Instead, growth responses were strongest in site 12 for the five species represented in that site (which had a moderate increase in light availability, but high base-line soil nutrient levels). Overall, seedlings (particularly of the shade intolerant species) in the moderately and severely defoliated sites generally attained a larger height in 2012 than seedlings in sites that were lightly defoliated (Fig 4). Seedlings attained the greatest (or equally greatest) height in site 12 (Fig 4).

**Table 2:** Linear mixed-effects model results of seedling height growth as a function of site, year and seedling height at the start of the growing season for six northern hardwood species. Per species, only models within two AIC_c_ (Akaike’s Information Criterion) units from the best model are included. The deviation from the best model in AIC_c_ units (ΔAIC_c_) is indicated. *n* indicates the number of included seedlings per species. The marginal (m) *R*^2^ (fixed effects only), and the conditional (c) *R*^2^ (both fixed and random effects) are indicated. acru = *Acer rubrum*; acsa = *Acer saccharum*; fagr = *Fagus grandifolia*; fram = *Fraxinus americana*; osvi = *Ostrya virginiana*; prse = *Prunus serotina*.

Species	*n*	ΔAIC_c_	*R*^*2*^(m)	*R*^*2*^(c)	Main effects	Interactions
acru	54	0.00	0.34	0.56	site, year, height	site × year
acru	54	0.57	0.35	0.55	site, year, height	site × year, site × height
acru	54	0.67	0.34	0.53	site, year	site × year
acru	54	1.98	0.36	0.54	site, year, height	site × year, year × height
acsa	212	0.00	0.30	0.40	site, year, height	site × year, year × height
fagr	87	0.00	0.28	0.48	site, year, height	site × year
fagr	87	1.44	0.29	0.48	site, year, height	site × year, site × height
fram	93	0.00	0.40	0.45	site, year, height	site × year, year × height
osvi	32	0.00	0.59	0.67	site, year, height	site × year, year × height
prse	34	0.00	0.27	0.33	site, year, height	

## Discussion

### Changes in canopy openness and soil resources

Not surprisingly, canopy openness strongly increased in response to forest tent caterpillar defoliation, consistent with hypothesis 1. Soil nitrogen (nitrate, ammonium and total inorganic nitrogen) also increased more strongly in sites with higher defoliation, consistent with hypothesis 1, and some previous studies (e.g., [15]). In contrast, Christenson et al. [47] and Frost and Hunter [48] found no change in soil total inorganic nitrogen concentration, perhaps because nitrogen from insect frass may become immobilized through microbial activity [49]. Nevertheless, some insect frass nitrogen can be directly incorporated into leaves in the same growing season [48]. In any case, the enhanced nutrient availability that we observed could influence seedling growth during an outbreak or in the subsequent year. Increased soil nitrogen concentrations may have important implications for water quality, as nitrate leakage from the system may be strong during, and after, an insect outbreak [50,51].

### Seedling growth responses to the forest tent caterpillar outbreak

Seedling growth strongly increased in 2009 in moderately (and severely) defoliated sites for two out of six species and in 2010 for all six species, supporting hypothesis 2. Thus, our results suggest that seedling growth increased in response to enhanced availability of light and soil resources. This is consistent with results of previous studies that found that seedling or sapling growth responds to light availability [52–55], soil resources [56] or to both light and soil resources [17,18,57]. It remains unclear whether the increase in canopy openness, the increase in soil nitrogen or both drove the increase in seedling growth, as 2009 and 2010 canopy openness and soil nitrogen levels were strongly correlated.

Other studies reported similar growth responses to defoliation. *Acer rubrum* saplings increased growth in plots defoliated by gypsy moth, but not in non-defoliated plots, in a hardwood forest in Connecticut [58]. In boreal forests, forest tent caterpillar outbreaks coincided with growth releases [59]. While we observed growth increases at defoliated sites, growth responses did not become stronger with the level of defoliation at a site. Growth responses under the highest canopy defoliation levels may have been constrained by defoliation of seedlings of some species or by nutrient limitation. Growth responses were most pronounced in the moderately defoliated, high-nutrient site 12. Thus, our results suggest that high soil nutrient levels facilitate a strong growth response to an increase in light, consistent with the idea that seedling growth responds more strongly to soil nutrients under high light [18,20,60,61].

There was a one-year lag in growth responses to forest tent caterpillar defoliation for four species. A lagged growth response could arise from determinate growth, i.e., environmental signals are set in the leaf primordia in the year prior to leaf out. Resource levels also remained higher in the year after the outbreak. We observed slightly higher canopy openness in 2010 than in non-outbreak conditions (2011), consistent with lower leaf area of defoliated trees the year after defoliation than in typical non-outbreak years [13], as well as higher soil nitrogen levels. Slight forest tent caterpillar defoliation in some sites in 2010 may have contributed to higher resource levels. In response to higher light, seedlings also shift allocation towards root growth [62] and non-structural carbohydrate storage [21], which may have resulted in a lagged growth response due to later mobilization of stored non-structural carbohydrates [21].

The strength of the growth response to the forest tent caterpillar outbreak varied strongly among species, with the strongest increase in the shade intolerant species *F*. *americana* and *P*. *serotina*, and in the shade tolerant species *O*. *virginiana*. These results are partly in agreement with hypothesis 3 that shade intolerant species respond more strongly to variation in light and nutrient availability than shade tolerant species [26,54,63]. The weak growth response in *A*. *saccharum* may be due to defoliation by forest tent caterpillar of part of the *A*. *saccharum* seedlings, and shading by seedlings of other species. In the severely defoliated site 11, the high density of larger *F*. *americana* seedlings decreased light availability to the smaller *A*. *saccharum* seedlings, and likely led to decreased *A*. *saccharum* growth during the outbreak [64]. The dense *F*. *americana* seedling layer may also explain the weaker growth increase of *F*. *americana* in site 11, as compared to site 12, despite the severe canopy defoliation in site 11.

### Insect outbreaks as a driver of forest dynamics

The forest tent caterpillar outbreak strongly increased light levels in the understory, soil nitrogen concentration and seedling growth. Shade intolerant species responded strongly to the outbreak: for these species recurring forest tent caterpillar outbreaks may facilitate understory persistence in the absence of canopy gaps. Fast early growth may increase the chance for seedlings to survive [8,65], and to eventually attain the forest canopy [66]. In addition, seedlings might be less susceptible to herbivory under the high-light conditions during the forest tent caterpillar outbreak through well-defended leaves [67]. Although not directly addressed in this study, insect outbreaks may also further facilitate regeneration of shade intolerant species by accelerating gap dynamics through elevated mature tree mortality of host species [68–70]. However, it is not known whether the seedlings that benefited from the forest tent caterpillar defoliation in our study, particularly the shade intolerant species, will persist in the understory until the next disturbance and resource enhancement. Seedling mortality resulting from low light, physical damage or herbivory [64] may still occur. Deer browsing, for example, may strongly reduce seedling survival [71], and therefore preclude longer-term growth and survival advantages of forest tent caterpillar defoliation. Increased canopy openness as a result of cyclical canopy defoliation by forest tent caterpillar may also be beneficial for other taxa. For example, higher abundance and/or species richness of invertebrates like butterflies [72–74] and spiders [75–77] is generally associated with a more open canopy. Thus, persistence of such invertebrate species in closed-canopy forests might also be facilitated by insect outbreaks. Overall, our results suggest that recurrent canopy defoliation resulting from cyclical forest insect outbreaks may be an additional driver of temperate forest dynamics, particularly in forests with a closed canopy.

## Supporting Information

S1 File Canopy openness data. Percent canopy openness for each of the six defoliated sites from 2009 to 2011. Quadrat indicates the number of the 1-m^2^ quadrat in the 200 m × 1 m transect.
(XLSX)

S2 File Soil resource data. Changes in soil soil nitrate (NO3), ammonium (NH4), and total inorganic nitrogen (TOTN) levels for each of the six defoliated sites from 2009 to 2011. The average change (from 2011 to either 2009 or 2010) in nondefoliated sites is indicated for each of the soil nutrients, as well as the change from 2011 to either 2009 or 2010 in the defoliated site itself. Quadrat indicates the number of the 1-m^2^ quadrat in the 200 m × 1 m transect.
(XLSX)

S3 File Seedling growth data. Seedling height growth, and seedling height at the start of the growing season, in the six defoliated sites from 2008 to 2012 for six northern hardwood species. Tag refers to the tag number of an individual seedling.
(XLSX)
